# Association of Estimated Pulse Wave Velocity With Survival: A Secondary Analysis of SPRINT

**DOI:** 10.1001/jamanetworkopen.2019.12831

**Published:** 2019-10-09

**Authors:** Charalambos Vlachopoulos, Dimitrios Terentes-Printzios, Stephane Laurent, Peter M. Nilsson, Athanase D. Protogerou, Konstatinos Aznaouridis, Panagiotis Xaplanteris, Iosif Koutagiar, Hirofumi Tomiyama, Akira Yamashina, Petros P. Sfikakis, Dimitrios Tousoulis

**Affiliations:** 1 Hypertension and Cardiometabolic Unit, First Department of Cardiology, Hippokration Hospital, Medical School, National and Kapodistrian University of Athens, Athens, Greece; 2 Department of Pharmacology, European Georges Pompidou Hospital, Assistance Publique Hôpitaux de Paris, Inserm UMR 970, University Paris Descartes, Paris, France; 3 Department of Clinical Sciences, Lund University, University Hospital, Malmö, Sweden; 4 Cardiovascular Prevention and Research Unit, Clinic and Laboratory of Pathophysiology, Laiko Hospital, Medical School, National and Kapodistrian University of Athens, Athens, Greece; 5 Division of Preemptive Medicine for Vascular Damage, Department of Cardiology, Tokyo Medical University, Tokyo, Japan; 6 First Department of Propaedeutic Internal Medicine, Medical School, Laiko General Hospital, National and Kapodistrian University of Athens, Athens, Greece

## Abstract

**Importance:**

Aortic stiffness, as assessed by carotid-femoral pulse wave velocity, is an independent predictor of future events in individuals with hypertension. Recent data suggest a predictive role of estimated pulse wave velocity (ePWV) calculated by previously published equations using age and blood pressure in future events in individuals with hypertension.

**Objective:**

To investigate whether ePWV and its response to treatment predict survival in the Systolic Blood Pressure Intervention Trial (SPRINT).

**Design, Setting, and Participants:**

This exploratory, hypothesis-generating, post hoc secondary analysis conducted from October 1, 2018, to August 31, 2019, examined data from 9361 participants in SPRINT and calculated ePWV at baseline and at 12 months. Adjusted hazard ratios (HRs) with 95% CIs of ePWV per 1 SD were estimated using Cox proportional hazards regression models. A total of 8450 patients were assigned to 4 groups according to their treatment allocation and their response in ePWV after 12 months.

**Interventions:**

Participants were assigned a systolic blood pressure target of less than 120 mm Hg (intensive treatment) or less than 140 mm Hg (standard treatment).

**Main Outcomes and Measures:**

The primary composite cardiovascular outcome was myocardial infarction, other acute coronary syndromes, stroke, heart failure, or death from cardiovascular causes.

**Results:**

In the SPRINT population (3332 women and 6029 men; mean [SD] age, 67.9 [9.4] years), ePWV predicted the primary outcome (HR, 1.30 [95% CI, 1.17-1.43]; *P* < .001) and all-cause death (HR, 1.65 [95% CI, 1.46-1.86]; *P* < .001) independent of the Framingham Risk Score. Estimated pulse wave velocity improved the C statistic model for the primary outcome from 0.676 (95% CI, 0.65-0.70) to 0.683 (95% CI, 0.66-0.71; *P* = .049) and improved the C statistic model for all-cause death from 0.67 (95% CI, 0.64-0.69) to 0.69 (95% CI, 0.66-0.72; *P* = .03). Net reclassification index indicated improvement in risk discrimination for survival compared with the Framingham Risk Score (categorical net reclassification index = 0.111; *P* < .001). Regarding response to treatment, intensive treatment was superior to standard treatment only when it was accompanied with a response in ePWV at the first year, while, within the standard treatment group, individuals whose ePWV responded to antihypertensive treatment had improved all-cause mortality, with a 42% lower risk of death compared with nonresponders (HR, 0.58 [95% CI, 0.36-0.94]; *P* = .03); effects were independent of changes in systolic blood pressure.

**Conclusions and Relevance:**

These results suggest that, in the SPRINT trial, ePWV predicted outcomes independent of the Framingham Risk Score, indicating an incremental role of markers of aortic stiffness on cardiovascular risk. Better survival of individuals whose ePWV responded to antihypertensive treatment independently of systolic blood pressure reduction suggests a role of markers of aortic stiffness as effective treatment targets in individuals with hypertension.

## Key Points

**Question:**

Do estimated markers of aortic stiffness, such as estimated pulse wave velocity and their change with time, predict cardiovascular events in individuals with hypertension?

**Findings:**

The results of this post hoc analysis of the randomized Systolic Blood Pressure Intervention Trial (SPRINT) support an incremental predictive role of estimated pulse wave velocity with outcomes beyond Framingham Risk Score. Individuals whose estimated pulse wave velocity responded to 1 year of antihypertensive treatment demonstrated a 42% lower risk of death compared with nonresponders independent of systolic blood pressure reduction in the standard treatment group of the Systolic Blood Pressure Intervention Trial.

**Meaning:**

Aortic stiffness could be used in individuals with hypertension to assess risk; it could be used also as a therapeutic target to assist patient management and improve prognosis.

## Teaser

This secondary analysis of the Systolic Blood Pressure Intervention Trial (SPRINT) investigates whether aortic stiffness, as assessed by estimated pulse wave velocity, and its response to treatment are associated with survival in individuals with hypertension.

## Introduction

Cardiovascular disease (CVD) is one of the major causes of death worldwide, and increased blood pressure (BP) is the most potent modifiable risk factor for CVD.^1^ Most risk prediction scores for future cardiovascular events, such as the Framingham Risk Score (FRS) and the Systematic Coronary Risk Evaluation (SCORE), incorporate BP in their variables.^2^ However, because of the scores’ need for simplicity, their overall predictive performance is suboptimal.^3^ This limitation has guided research toward the investigation and implementation of vascular biomarkers associated with or influenced by hypertension to fill in the gap of missed residual risk as well as to improve CVD risk individualization.

In hypertension, arteries represent an organ with damage from elevated BP^4,5^; however, arterial stiffening is also considered to be a causal factor leading to hypertension because it precedes and predicts the incidence of the latter.^6^ The most robust and well-studied marker of aortic stiffness, the carotid-femoral pulse wave velocity (cfPWV), has shown an incremental predictive value for future cardiovascular events and all-cause mortality beyond classical risk scores and BP, which is the main modifiable determinant of aortic stiffness.^7,8^ Moreover, there is limited evidence suggesting that elevated cfPWV is associated with the poor response of BP to BP-lowering drugs^9^ and that cfPWV regression is associated with improved survival.^10^

Measurement of cfPWV is well standardized, noninvasive, simple, and easy^11^: it requires the use of specific devices that have not extensively infiltrated clinical practice.^12,13^ Although important scientific bodies have endorsed the use of cfPWV in clinical practice with varying degrees of recommendation,^1,12,13^ there is agreement that simplification of the technology and research into new inexpensive methods to measure or estimate aortic stiffness will facilitate its adoption in clinical practice. Toward this end, there have been several efforts of estimating aortic stiffness either through equations including age and mean BP (MBP)^14^ or using artificial intelligence.^15^ The latter takes an uncalibrated trace of carotid pressure waveform and performs intrinsic frequency analysis and processes the signal. Then, it combines the results of these analyses with traditional clinical variables, such as age, and models the PWV by neural networks through bootstrap averaging.^15^ These estimates of cfPWV have shown strong correlations (linear *R*^2^ = 0.45 in patients with cardiovascular risk factors) with in vivo measurements, and estimated PWV (ePWV) has shown a predictive role compared with traditional risk scores, especially in patients with untreated hypertension.^14^

Therefore, the aims of this study were to (1) investigate whether ePWV predicted the primary outcome and all-cause death in the participants of the landmark Systolic Blood Pressure Intervention Trial (SPRINT) independently of the FRS and BP, (2) investigate whether ePWV improved the risk prediction significantly when the FRS and BP were included in the model, and (3) investigate whether regression of arterial stiffness, defined as response (effective lowering) of ePWV to antihypertensive treatment in the 2 treatment groups (ie, intensive vs standard systolic BP [SBP] goal) at 12 months, predicted the primary outcome and all-cause death when the FRS and BP response were included in the model.

## Methods

### Study Population

This exploratory, hypothesis-generating, post hoc secondary analysis conducted from October 1, 2018, to August 31, 2019, examined data from 9361 participants in SPRINT, a multicenter, randomized, open-label, controlled 2-group trial conducted in patients at increased risk for CVD (based on a history of clinical or subclinical CVD, chronic kidney disease, a 10-year Framingham general CVD risk ≥15%, or age ≥75 years). The protocol, the baseline characteristics, and the main results of the study have been published.^16^ A total of 9361 participants were enrolled between November 11, 2010, and March 15, 2013. Patients were randomized to an SBP target of less than 120 mm Hg (intensive treatment) or a target of less than 140 mm Hg (standard treatment). The primary outcome was a composite of nonfatal myocardial infarction, acute coronary syndrome not resulting in myocardial infarction, nonfatal stroke, nonfatal acute decompensated heart failure, and death from cardiovascular causes. Secondary outcomes included the individual components of the primary composite outcome, death from any cause, and the composite of the primary outcome or death from any cause. Definition and adjudication procedures of outcome events have been published.^16^ The median follow-up in 2015 when the study was ended was 3.26 years. Study design and reporting were based on the Transparent Reporting of a Multivariable Prediction Model for Individual Prognosis or Diagnosis (TRIPOD) reporting guideline, a standardized, evidence-based set of recommendations for reporting prediction modeling studies.^17^ The Hippokration Hospital Research Ethics Committee deemed this analysis exempt from review and waived the need for obtaining informed patient consent because the data were deidentified.

### Calculation of ePWV

Using the equation described in the study by Greve et al^14^ that was derived by the Reference Values for Arterial Stiffness’ Collaboration,^18^ ePWV was calculated from age and MBP: ePWV = 9.587 − 0.402 × age + 4.560 × 10^−3^ × age^2^ − 2.621 × 10^−5^ × age^2^ × MBP + 3.176 × 10^−3^ × age × MBP − 1.832 × 10^−2^ × MBP. Mean BP was calculated as diastolic BP (DBP) + 0.4(SBP − DBP). For patients in SPRINT, the equation used to calculate ePWV was the one derived from the reference population (individuals or patients of both sexes presenting CVD risk factors that had been shown to have no independent influence on cfPWV values) because all of the participants in SPRINT had at least 1 CVD risk factor.

### Statistical Analysis

Data are presented as mean and SD for continuous variables, as median value (25th-75th percentiles) for skewed variables, and as numbers and percentages for categorical variables.

For all clinical end points, the study-adjusted hazard ratios (HRs) with 95% CIs of ePWV per 1 SD were estimated using Cox proportional hazards regression models with 2-sided tests at a *P* < .05 level of significance. We also derived the noncardiovascular death outcome by removing cardiovascular deaths from the all-cause death end point. We decided to further investigate all-cause mortality (ie, the most clinically meaningful outcome) and the primary outcome, either on their own or as a combination, as predefined in SPRINT. The model included the FRS, assigned treatment (intensive vs standard), the presence of clinical or subclinical CVD, antihypertensive treatment at baseline, and SBP at baseline. Whether adding ePWV to the Cox proportional hazards regression model was successful was tested using the likelihood ratio test.

Model discrimination was assessed with the C statistic. The ability of ePWV to reclassify individuals with hypertension into a different mortality risk category was tested using the net reclassification index (NRI). The Cox proportional hazards regression models were stratified for the model, including FRS risk in 4 categories. Patients were classified as being at low cardiovascular risk (10-year risk of cardiovascular events, <10%), low to intermediate cardiovascular risk (10-year risk of cardiovascular events, 10% to <15%), high to intermediate cardiovascular risk (10-year risk of cardiovascular events, 15% to <20%), or high cardiovascular risk (10-year risk of cardiovascular events, ≥20%). We calculated 2 versions of the NRI: a categorical NRI (catNRI) based on the aforementioned categories and a category-free (continuous) NRI (contNRI), which is independent of arbitrarily defined risk thresholds. The integrated discrimination improvement index was also estimated (eAppendix 1 in the Supplement).

### Effective ePWV Response to Treatment

We also assessed the effect of 12 months of antihypertensive treatment on ePWV. We categorized 8450 patients at 12 months (after excluding 3 patients who were lacking SBP measurements at their 12-month visits) into responders to treatment regarding aortic stiffness if their change in ePWV (ΔePWV = ePWV at 12 months – ePWV at baseline) at 12 months was 0.15 m/s or less; all patients with ΔePWV greater than 0.15 m/s were classified as nonresponders. The cutoff of 0.15 m/s was chosen based on the expected annual change of PWV of patients receiving antihypertensive treatment.^5,19^ Our approach takes into account not only any change according to treatment (ie, decrease of ePWV) but also the expected change owing to aging (ie, increase of ePWV). Furthermore, we categorized patients into 4 groups according to their treatment allocation and whether there was an effective response in ePWV after 12 months of antihypertensive treatment or not (group 1 [n = 1913], ePWV nonresponders receiving standard treatment; group 2 [n = 2307], ePWV responders receiving standard treatment; group 3 [n = 684], ePWV nonresponders receiving intensive treatment; and group 4 [n = 3546], ePWV responders receiving intensive treatment). The association of the groups with all-cause death or the primary outcome was assessed using Cox proportional hazards regression models in which all of the covariates in the model plus ePWV were included in the model as standard covariates. To further assess the effect of changes in BP during treatment, we adjusted for change in SBP at 12 months (SBP at 12 months − SBP at baseline). We also performed a subgroup analysis of the response to ePWV based on the allocation of treatment (standard or intensive). Patients free of events at 12 months were included in the analyses. Data analysis was performed with SPSS software, version 20 (SPSS Inc) and Stata software, version 13.0 (StataCorp LP).

## Results

When tested in the 9361 patients (3332 women and 6029 men; mean [SD] age, 67.9 [9.4] years) of the SPRINT population (Table 1; eAppendix 2 and eFigures 1-3 in the Supplement), ePWV was associated with all-cause death (HR, 1.65; 95% CI, 1.46-1.86; *P* < .001), the primary outcome (HR, 1.30; 95% CI, 1.17-1.43; *P* < .001), stroke (HR, 1.45; 95% CI, 1.20-1.76; *P* < .001), heart failure (HR, 1.70; 95% CI, 1.42-2.04; *P* < .001), cardiovascular death (HR, 1.39; 95% CI, 1.10-1.76; *P* = .006), and noncardiovascular death (HR, 1.76; 95% CI, 1.53-2.03; *P* < .001) (Table 2) independent of the FRS and other relevant confounders, even after adjustment for baseline SBP or MBP (for analysis with MBP, see eAppendix 2 in the Supplement). We also assessed whether the predictive ability of ePWV persisted after 12 months of antihypertensive treatment. Similarly, ePWV at 12 months was predictive of the same future outcomes as baseline ePWV (HR for all-cause death, 1.50; 95% CI, 1.31-1.72; *P* < .001; and HR for primary outcome, 1.23; 95% CI, 1.11-1.35; *P* < .001).

**Table 1.** Baseline Characteristics of Participants in SPRINT

Characteristic	Value (N = 9361)^a^
Female	3332 (35.6)
Age, mean (SD), y	67.9 (9.4)
Black race	2947 (31.5)
Blood pressure, mean (SD), mm Hg	
Systolic	139.7 (15.6)
Diastolic	78.1 (11.9)
Mean	102.7 (11.5)
Pulse	61.5 (14.4)
Estimated pulse wave velocity, mean (SD), m/s	11.3 (1.7)
Framingham Risk Score, mean (SD), %^b^	24.8 (12.5)
Cardiovascular disease, clinical or subclinical	1877 (20.1)
Chronic kidney disease	2646 (28.3)
Estimated GFR, mean (SD), mL/min/1.73 m^2^	71.7 (20.6)
Ratio of urinary albumin, mg, to creatinine, g, median (IQR)	9.5 (5.6-21.4)
Fasting total cholesterol, mean (SD), mg/dL	190.1 (41.2)
Fasting HDL cholesterol, mean (SD), mg/dL	52.9 (14.5)
Fasting total triglycerides, median (IQR), mg/dL	107.0 (77.0-150.0)
Fasting plasma glucose, mean (SD), mg/dL	98.8 (13.5)
Current smokers	1240 (13.2)
Body mass index, mean (SD)^c^	29.9 (5.8)
Antihypertensive agents, mean (SD), No. per patient	1.8 (1.0)
Not using antihypertensive agents	882 (9.4)

Abbreviations: GFR, glomerular filtration rate; HDL, high-density lipoprotein; IQR, interquartile range; SPRINT, Systolic Blood Pressure Intervention Trial.

SI conversion factors: To convert total cholesterol and HDL cholesterol to millimoles per liter, multiply by 0.0259; triglycerides to millimoles per liter, multiply by 0.0113; and glucose to millimoles per liter, multiply by 0.0555.

^a^ Data are presented as number (percentage) of participants unless otherwise indicated.

^b^ Calculated for 9312 participants with available data.

^c^ Calculated as weight in kilograms divided by height in meters squared.

**Table 2.** Association of ePWV With Different End Points in the Patients With Hypertension From SPRINT^a^

Outcome	No.	HR per 1 SD of ePWV (95% CI)	*P* Value
Primary outcome	558	1.30 (1.17-1.43)	<.001
MI	212	1.10 (0.94-1.30)	.24
Non-MI acute coronary syndrome	80	1.03 (0.78-1.36)	.83
Stroke	131	1.45 (1.20-1.76)	<.001
Heart failure	162	1.70 (1.42-2.04)	<.001
CV death	100	1.39 (1.10-1.76)	.006
Death from non-CV cause	262	1.76 (1.53-2.03)	<.001
All-cause death	362	1.65 (1.46-1.86)	<.001
Primary outcome or death	750	1.36 (1.25-1.48)	<.001

Abbreviations: CV, cardiovascular; ePWV, estimated pulse wave velocity; HR, hazard ratio; MI, myocardial infarction; SPRINT, Systolic Blood Pressure Intervention Trial.

^a^ The model includes Framingham Risk Score, antihypertensive assigned treatment (intensive vs standard), the presence of clinical or subclinical CV disease, antihypertensive treatment at baseline, and systolic blood pressure at baseline and was assessed separately for each outcome.

**Figure 1.**
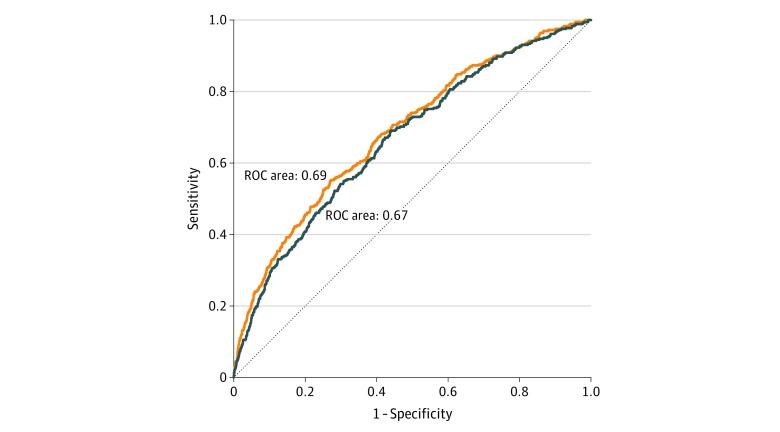
Receiver Operating Characteristic (ROC) Curves for Prediction of All-Cause Death A comparison of the model including the Framingham Risk Score (blue curve) with the model including the Framingham Risk Score and estimated pulse wave velocity (orange curve).

Furthermore, ePWV modestly improved the Cox proportional hazards regression models, including the covariates of the model for all 3 end points of the study (primary outcome, all-cause death, and primary outcome or death) (Figure 1; eTable 1 and eFigure 4 in the Supplement). The model for all-cause death without ePWV resulted in a likelihood ratio χ^2^ = 142.8 (*P* < .001). Addition of ePWV to the model successfully modestly improved the model (likelihood ratio, χ^2^ = 205.8; *P* < .001 vs model without ePWV). Specifically, addition of ePWV modestly improved the C index from 0.67 (95% CI, 0.64-0.69) to 0.69 (95% CI, 0.66-0.72; *P* = .03). Correspondingly, the addition of ePWV modestly improved the C index of the model from 0.676 (95% CI, 0.65-0.70) to 0.683 (95% CI, 0.66-0.71; *P* = .049) for the primary outcome.

Estimated carotid-femoral pulse wave velocity reclassified patients with risk of all-cause death into a different mortality risk category, with a statistically significant catNRI for model categories (catNRI = 0.111; 95% CI, 0.066-0.156; *P* < .001; eTable 2 in the Supplement) and contNRI of 0.36 (95% CI, 0.25-0.45; *P* < .001) compared with the model including the FRS. Furthermore, the estimated integrated discrimination improvement index was 0.0096 (*P* < .001). Similarly, improvement of catNRI and contNRI was statistically significant for the primary outcome (catNRI = 0.055 [95% CI, 0.021-0.090]; *P* = .002; and contNRI = 0.22 [95% CI, 0.12-0.30]; *P* < .001) and the primary outcome and death (catNRI = 0.038 [95% CI, 0.002-0.074]; *P* = .04; and contNRI = 0.25 [95% CI, 0.16-0.33]; *P* < .001). Moreover, the estimated integrated discrimination improvement index for the primary outcome was 0.002 (*P* = .003) and for the end point of primary outcome or death was 0.005 (*P* < .001) (for analysis with MBP, see eAppendix 2 in the Supplement).

### Response of ePWV to Treatment

We observed a reduction of mean (SD) ePWV in the intensive treatment group but no change in the standard treatment (−0.75 [0.98] vs 0.03 [0.95] m/s; *P* < .001) (eFigure 5 in the Supplement). Furthermore, the proportion of participants whose ePWV responded to antihypertensive treatment was larger in the intensive treatment group compared with the standard treatment group (3546 of 4230 [83.8%] vs 2307 of 4220 [54.7%]; *P* < .001; eFigure 6 in the Supplement). Associations of the response of ePWV with the 2 end points, independent of change in SBP (eAppendix 2 and eTable 3 in the Supplement), were as follows.

#### Primary Outcome

After adjusting for all of the covariates in the model plus baseline ePWV and change in SBP for the primary outcome (345 events), we observed that the group receiving intensive treatment with a concomitant reduction in ePWV (group 4) had the best prognosis beyond the 12 months of treatment (eFigure 7 in the Supplement). More important, the benefit of intensive treatment compared with standard treatment on the primary outcome was observed only for those whose ePWV responded to treatment rather than nonresponders. Groups 1 and 2 (standard treatment groups) had similar risk for the primary outcome as those in the intensive treatment group whose ePWV did not respond to treatment (group 1 vs group 3: HR, 0.79; 95% CI, 0.51-1.23; *P* = .30; and group 2 vs group 3: HR, 0.85; 95% CI, 0.53-1.36; *P* = .50). In a subgroup analysis, there were no statistically significant differences between ePWV responders and nonresponders in the standard treatment group or in the intensive treatment group.

#### All-Cause Death

**Figure 2.**
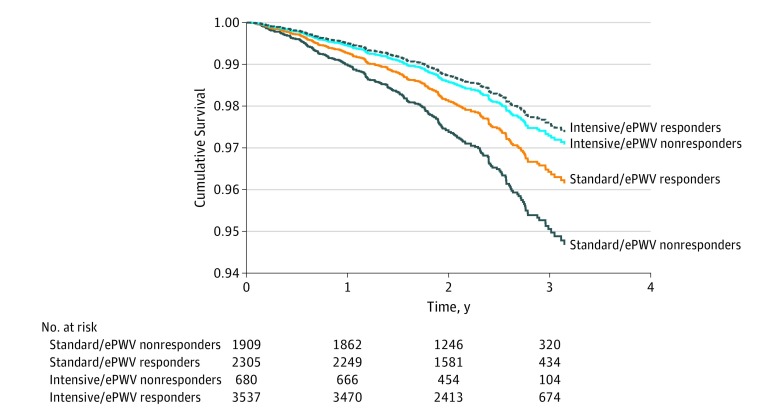
Combined Effect of Treatment Allocation and Response of Estimated Pulse Wave Velocity (ePWV) to Treatment on All-Cause Death Time zero is 12 months after randomization.

**Figure 3.**
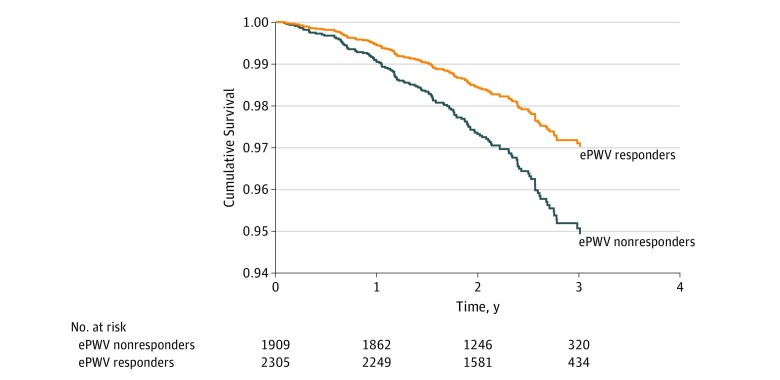
Effect of the Response of Estimated Pulse Wave Velocity (ePWV) to 12 Months of Treatment on All-Cause Death in the Standard Treatment Group Time zero is 12 months after randomization. The hazard ratio for ePWV responders is 0.58 (95% CI, 0.36-0.94) (*P* = .03).

After adjusting for all of the covariates in the model plus baseline ePWV and change in SBP, we observed that the group receiving intensive treatment with a concomitant reduction in ePWV (group 4) had the best prognosis concerning all-cause death events (240 events) beyond the 12 months of treatment (Figure 2). More important, the beneficial effect of intensive treatment was attenuated for those whose ePWV did not respond to treatment because, although survival was higher compared with nonresponders in the standard treatment group (HR, 0.54; 95% CI, 0.30-0.97; *P* = .04), survival was similar to those in the standard treatment group as long as the latter were ePWV responders (HR, 0.75; 95% CI, 0.41-1.40; *P* = .37). Furthermore, we observed a difference in the effect of ePWV response between the 2 treatment groups. We performed a subgroup analysis and observed that, in the standard treatment group independent of change in SBP (for analysis with MBP, see eAppendix 2 in the Supplement), responders had a lower risk compared with nonresponders (HR, 0.58; 95% CI, 0.36-0.94; *P* = .03) (Figure 3). This was not apparent in the intensive treatment group (HR, 1.38; 95% CI, 0.66-2.89; *P* = .39). More important, baseline ePWV was a significant independent predictor of death in both the intensive treatment group (HR, 1.78; 95% CI, 1.47-2.15; *P* < .001) and standard treatment group (HR, 1.55; 95% CI, 1.23-2.00; *P* < .001).

## Discussion

To our knowledge, the present analysis provides the largest population of individuals with hypertension to investigate the possible incremental predictive role of ePWV as well as its use as a therapeutic target. We demonstrate an independent predictive role of ePWV for most clinical end points of SPRINT and an improved predictive ability beyond the FRS regarding all-cause mortality and/or the primary outcome. More important, intensive treatment was superior to standard treatment only when it was accompanied with a response in ePWV at the first year, while, within the standard group, those with an ePWV response had improved all-cause mortality. These effects were independent of SBP reduction and support a potential role of markers of aortic stiffness as effective treatment targets in patients with hypertension.

### Integration of Vascular Aging in Clinical Practice

Early vascular aging rather than chronological aging can conceptually offer better risk prediction.^20^ Carotid-femoral pulse wave velocity, at present the most widely studied index of arterial stiffness, fulfills most of the stringent criteria for a clinically useful biomarker.^13^ Carotid-femoral pulse wave velocity is reproducible, accurate, and easy to measure in a noninvasive manner according to a well-defined protocol, and the obtained metric distinguishes individuals at risk with significant reclassification into a different mortality risk category, especially for those at intermediate risk (13% for 10-year CVD risk) and in younger individuals.^8^ Therefore, cfPWV is contemporarily deemed suitable for use in clinical practice.^13^ Accumulation of classical risk factors leads to acceleration of early vascular aging.^5,19,20,21^ Specifically for hypertension, it represents not only disease-mediated organ damage but also a predictor of its development.^6^ However, despite numerous recommendations by European guidelines^13^ and an American Heart Association scientific statement,^22^ its use in clinical practice is suboptimal, mainly owing to practical and logistic reasons.

Although reimbursement from health care authorities and reduction of the cost of dedicated devices can lead to further use of PWV measurement, parallel efforts for effective integration into clinical practice have been offered. One method using a simple clinical score (SAGE [SBP, age, glycemia, and estimated glomerular filtration rate] score) that predicts high cfPWV values on the basis of widely available clinical variables can prioritize measurement of cfPWV.^23^ The other method is based on the determination of reference values for cfPWV^18^: estimated cfPWV is derived by relevant equations that take into account age and BP. Despite its great dependence on these 2 parameters, ePWV was shown to be predictive of future cardiovascular events and improved risk prediction compared with traditional risk scores, such as the FRS and SCORE, mainly in healthy individuals and those with untreated hypertension.^14,24^ Although the first approach^23^ aims at judicial use of existing resources and the second (ePWV) is applicable in their absence, they both can result in greater accumulation of evidence and appreciation of the clinical role of aortic stiffness.

### Estimated PWV

#### Previous Knowledge

Our study confirmed the initial report by Greve et al^14^ of the incremental role of ePWV in risk prediction and especially in risk prediction of death. Despite the fact that ePWV has not been previously estimated in the SPRINT population, cfPWV has been measured in a subset of elderly SPRINT participants.^25^ Although the predictive role was not investigated, there were discrepancies between cfPWV and ePWV that increased with levels of cfPWV. However, in that report, participants were not stringently classified according to levels of BP; had classification been done properly, the equations from the Reference Values for Arterial Stiffness’ Collaboration^18^ would apply in this specific SPRINT subpopulation.

#### Clinical Implications

Our findings may have important clinical implications. In a variety of tests, ePWV demonstrated a predictive ability beyond that of traditional risk scoring, such as the FRS. More important, 1 of 10 patients with hypertension were reclassified into a different mortality risk category by ePWV. Taken together, these findings suggest that ePWV and the FRS, despite including age and BP, do not impart the same risk information owing to both inadequacies of the FRS equation to assess cardiovascular risk as well as the added value of ePWV. Thus, ePWV can be used to improve risk prediction in addition to traditional risk classification in conditions under which measuring cfPWV is not feasible. Furthermore, the use of ePWV will result in greater acknowledgment of the role of aortic stiffness and will aid physicians in implementing it in clinical practice.

Second, ePWV can be used to gauge the effect of treatment. Although, as shown in SPRINT, reduction of BP is the driving element of risk reduction, it appears that it is not the only element of risk reduction. When compared with standard treatment, intensive treatment was superior only when it was accompanied by a response in ePWV at the first year. The importance is further augmented when a reduction in BP is modest: within the standard treatment group, those with ePWV response had improved all-cause mortality. This result could translate into clinical practice in a dual manner, as it could inform the physician as to which patients must receive more intensive treatment and at the same time it could protect responders from a further increase in dose and number of antihypertensive regimens that may increase their treatment-related complications, as seen in SPRINT. These results must be confirmed in studies in which actual cfPWV is measured. Although the Strategy for Preventing Cardiovascular and Renal Events Based on Arterial Stiffness trial that addresses this issue is awaited,^26^ results from indirect approaches^10^ or from indices related to aortic stiffness, such as central pressures,^27,28^ were positive.

In addition to the predictive value for cardiovascular end points, an intriguing finding was the prediction of noncardiovascular deaths. This finding is not unexpected because all relevant survival studies, including several meta-analyses, have shown an extremely close relationship of cfPWV with all-cause mortality.^7,8,29^ Beyond having a causal effect on cardiovascular events, vascular aging may also reflect biological aging in general: certain pathophysiological pathways may affect both noncardiovascular conditions and aortic stiffness. Such links, however, are not readily available. Nevertheless, while aortic stiffness depends largely on BP, the former is a major predictor of all-cause mortality, and angiogenic indices of hypertension, such as angiotensin II, vascular endothelial growth factor, and oxidative stress, have been linked to the development of cancer.^30^ Furthermore, aortic stiffness has been linked etiologically to inflammation and oxidative stress,^24,31^ which in turn participate in the pathophysiological characteristics of diseases that carry increased fatality, such as cancer and chronic inflammatory diseases. Moreover, there is a strong association of vascular biomarkers with genetic markers of biological aging and life expectancy, such as telomere length, implying a genetic common predisposition of arterial function and death.^32,33^

### Strengths and Limitations

The major strength of our study is the use of data from a well-organized study with close follow-up of patients. Although aspects of this trial have been criticized,^34,35^ it has been influential on current recommendations.^12,36^ Furthermore, its main findings were corroborated from large meta-analyses of randomized clinical trials showing that intensive BP reduction decreases cardiovascular events.^37,38^ Our models were adjusted for baseline risk as well as BP.

Duration of follow-up was modest, reaching approximately 3 years. However, the number of outcome events was adequate for the study to be powered for a multitude of end points, including mortality. The present analysis is a post hoc one restricted by its inherent limitations. Nevertheless, most of the principal findings of SPRINT were confirmed (albeit as a trend in some findings owing to the relatively increased 95% CIs in our population).

We cannot exclude that chance owing to multiple testing could have played a part in our results, especially for the end points of the subgroup analysis. SPRINT was not powered to examine individual components of the primary outcome. For these reasons, we did not analyze all outcomes reported in SPRINT and focused on the 2 major and clinically meaningful outcomes.

SPRINT excluded patients with diabetes or history of stroke. This fact may limit the generalizability of our findings to those patients. However, SPRINT included a large, diverse population of individuals with hypertension at high risk for cardiovascular events that represents a major proportion of the population visiting hypertension clinics.

Our results were based exclusively on the FRS. More contemporary risk scores, such as the pooled cohort’s 10-year atherosclerotic CVD (ASCVD) risk score and SCORE, could be alternative comparators to the predictive value of ePWV. However, both have been applied to populations that are mainly free of CVD with lower risk for CVD risk, contrary to the SPRINT population at high CVD risk. In addition, there are certain limitations regarding the age span (SCORE, 40-65 years; and ASCVD, 20-79 years) that would substantially decrease the available population for analysis in our case. Furthermore, SCORE has not been found to predict risk effectively in non-European populations and has not been calibrated to US individuals, while it has been shown to highly overestimate risk (>50%).^39^ Moreover, ASCVD demonstrated poor calibration and also significant overestimation of cardiovascular risk in SPRINT.^40^ For these reasons, we decided to rely only on the FRS, which is found to have the smallest overestimation of risk compared with the other 2 risk scores.

## Conclusions

The present post hoc analysis of the SPRINT data supports an incremental predictive role of ePWV as well as possible use of this marker as a therapeutic target in patients with hypertension. Estimated carotid-femoral pulse wave velocity predicts outcome beyond the FRS, and the better survival of responders to PWV independent of SBP reduction suggests a role for markers of aortic stiffness as effective treatment targets in patients with hypertension. These results reinforce the notion of markers of vascular aging and support studies with actual aortic stiffness measurements in prospective clinical trials.

## Notes

Supplement. **eAppendix 1.** Methods **eAppendix 2.** Results **eReferences** **eTable 1.** Cox Regression Models Predicting the Principal End Points in the SPRINT Trial and Adjusted for Model (Including Framingham Risk Score) Risk, With Estimated Pulse Wave Velocity (ePWV) **eTable 2.** Reclassification of the Predicted Risk of Death by ePWV When Added to Model (Including FRS) **eTable 3.** Hazard Ratios and 95% CI for the Comparison Between Responses to Treatment Groups in the Cox Regression Survival Analysis **eFigure 1.** Histograms of Estimated Pulse Wave Velocity Based on Sex **eFigure 2.** Histograms of Estimated Pulse Wave Velocity Based on Race and Age Groups **eFigure 3.** Correlation Between (A) Estimated Pulse Wave Velocity and Systolic Blood Pressure and (B) Between Their 12-Month Changes **eFigure 4.** Receiver Operating Curves for the Primary Outcome and All-Cause Death **eFigure 5.** The Change of Estimated Pulse Wave Velocity at 12 Months in the Two Treatment Arms **eFigure 6.** Proportion of Estimated Pulse Wave Velocity (ePWV) Responders (Green Bars) Compared to Nonresponders (Blue Bars) in the Two Treatment Arms **eFigure 7.** The Combined Effect of Treatment Allocation and Response of Estimated Pulse Wave Velocity (ePWV) to Treatment on the Primary Outcome
