# Supplementary material for: Association of Estimated Pulse Wave Velocity With Survival: A Secondary Analysis of SPRINT
Source: JAMA Netw Open. 2019 Oct 9;2(10):e1912831. doi: 10.1001/jamanetworkopen.2019.12831 (PMC6802234; doi:10.1001/jamanetworkopen.2019.12831)
Supplement: Supplement. — eAppendix 1. Methods eAppendix 2. Results eReferences eTable 1. Cox Regression Models Predicting the Principal End Points in the SPRINT Trial and Adjusted for Model (Including Framingham Risk Score) Risk, With Estimated Pulse Wave Velocity (ePWV) eTable 2. Reclassification of the Predicted Risk of Death by ePWV When Added to Model (Including FRS) eTable 3. Hazard Ratios and 95% CI for the Comparison Between Responses to Treatment Groups in the Cox Regression Survival Analysis eFigure 1. Histograms of Estimated Pulse Wave Velocity Based on Sex eFigure 2. Histograms of Estimated Pulse Wave Velocity Based on Race and Age Groups eFigure 3. Correlation Between (A) Estimated Pulse Wave Velocity and Systolic Blood Pressure and (B) Between Their 12-Month Changes eFigure 4. Receiver Operating Curves for the Primary Outcome and All-Cause Death eFigure 5. The Change of Estimated Pulse Wave Velocity at 12 Months in the Two Treatment Arms eFigure 6. Proportion of Estimated Pulse Wave Velocity (ePWV) Responders (Green Bars) Compared to Nonresponders (Blue Bars) in the Two Treatment Arms eFigure 7. The Combined Effect of Treatment Allocation and Response of Estimated Pulse Wave Velocity (ePWV) to Treatment on the Primary Outcome [file jamanetwopen-2-e1912831-s001.pdf]

Supplementary Online Content

Vlachopoulos C, Terentes-Printzios D, Laurent S, et al. Association of estimated pulse wave velocity with survival: a secondary analysis of SPRINT. *JAMA Netw Open.* 2019;2(10):e1912831. doi:10.1001/jamanetworkopen.2019.12831

eAppendix 1. Methods
eAppendix 2. Results
eReferences
eTable 1. Cox Regression Models Predicting the Principal End Points in the SPRINT Trial and Adjusted for Model (Including Framingham Risk Score) Risk, With Estimated Pulse Wave Velocity (ePWV)
eTable 2. Reclassification of the Predicted Risk of Death by ePWV When Added to Model (Including FRS)
eTable 3. Hazard Ratios and 95% CI for the Comparison Between Responses to Treatment Groups in the Cox Regression Survival Analysis
eFigure 1. Histograms of Estimated Pulse Wave Velocity Based on Sex
eFigure 2. Histograms of Estimated Pulse Wave Velocity Based on Race and Age Groups
eFigure 3. Correlation Between (A) Estimated Pulse Wave Velocity and Systolic Blood Pressure and (B) Between Their 12-Month Changes
eFigure 4. Receiver Operating Curves for the Primary Outcome and All-Cause Death
eFigure 5. The Change of Estimated Pulse Wave Velocity at 12 Months in the Two Treatment Arms
eFigure 6. Proportion of Estimated Pulse Wave Velocity (ePWV) Responders (Green Bars) Compared to Nonresponders (Blue Bars) in the Two Treatment Arms
eFigure 7. The Combined Effect of Treatment Allocation and Response of Estimated Pulse Wave Velocity (ePWV) to Treatment on the Primary Outcome

This supplementary material has been provided by the authors to give readers additional information about their work.

© 2019 Vlachopoulos C et al. *JAMA Network Open.*

eAppendix 1. Methods

Measures of Discrimination

We used the “roccomp” procedure in Stata 13.0 to compute and compare the AUC of the model-based predicted probability of dying during follow-up with the observed outcome (death vs. survival). The traditional Net Reclassification Improvement (NRI)[1] requires clinically meaningful risk strata, therefore we used four categories (low [10-year risk of CV events < 10%], low-intermediate [10-year risk of CV events between 10% and< 15%], high-intermediate [10-year risk of CV events between 15% and< 20%] or high [10-year risk of CV events ≥20%] cardiovascular risk). We also used a newer category-free version, continuous NRI(>0),[2] which quantifies the correct movement of model-based probabilities when additional markers are added to the model: downward for survivors and upward for decedents.[3] An alternative measure, the Integrated Discrimination Improvement (IDI), can be interpreted as the difference in discrimination slopes of models with and without the new markers, where the discrimination slope is the absolute difference in the average prediction between those who experienced the event and those who did not.[3] Although there are no established benchmarks, Pencina et al.[4] suggest ΔAUC>0.01 represents a meaningful improvement, while continuous NRI greater than 0.2 implies at least moderate improvement. Researchers do not provide a corresponding gauge for IDI.

© 2019 Vlachopoulos C et al. *JAMA Network Open.*

eAppendix 2. Results

There were no major differences in distribution between gender, age and race especially taking into consideration the different age and systolic blood pressure (SBP) in each group (eFigure 1 and 2). As expected there was a correlation between estimated pulse wave velocity (ePWV) and SBP ($r=0.497$, $p<0.001$; eFigure 3A). Furthermore, the correlation between ΔSBP and ΔePWV was much higher ($r=0.948$, $p<0.001$; eFigure 3B).

Analysis of the predictive role of ePWV when adjusting for mean blood pressure (MBP)

ePWV predicted all-cause death, primary outcome, stroke, heart failure, cardiovascular death and non-cardiovascular death independently of FRS and other relevant confounders, even after adjustment for baseline MBP ($p\leq0.01$ for all).

Furthermore, ePWV significantly modestly improved the Cox regression models for both all-cause death and primary outcome. Specifically, addition of ePWV modestly improved the C index from 0.67 (95 % CI: 0.64–0.70) of Model to 0.69 (95 % CI: 0.66–0.72) with $p<0.05$ for all-cause death. Correspondingly, the addition of ePWV modestly improved the C index from 0.676 (95 % CI: 0.652–0.699) of the Model to 0.683 (95 % CI: 0.660–0.706) with $p<0.05$ for the primary outcome. ePWV reclassified hypertensive patients for all-cause death with a statistically significant categorical NRI (catNRI) for Model categories (catNRI=0.113, $p<0.001$). Furthermore, the estimated IDI was 0.009 ($p<0.001$). Similarly, improvement of catNRI was statistically significant for the primary outcome (catNRI=0.045, $p=0.007$) Moreover, the estimated IDI was 0.002 ($p=0.005$).

Regarding the response to treatment, results were similar when adjusted for MBP and change in MBP instead of SBP. Specifically, in the standard treatment arm independently of change in MBP, responders had a lower risk compared to non-responders (HR=0.49 [95% CI: 0.29-0.82]; $p=0.007$).

© 2019 Vlachopoulos C et al. *JAMA Network Open.*

eReferences

1. Pencina MJ, D'Agostino RB,Sr., D'Agostino RB,Jr., Vasan RS. Evaluating the added predictive ability of a new marker: from area under the ROC curve to reclassification and beyond. Stat Med 2008;27(2):157,72; discussion 207-12.
2. Pencina MJ, D'Agostino RB S, Steyerberg EW. Extensions of net reclassification improvement calculations to measure usefulness of new biomarkers. Stat Med 2011;30(1):11-21.
3. Goldman N, Glei DA. Quantifying the value of biomarkers for predicting mortality. Ann Epidemiol 2015;25(12):901,906.e4.
4. Pencina MJ, D'Agostino RB, Pencina KM, Janssens ACW, Greenland P. Interpreting incremental value of markers added to risk prediction models. Am J Epidemiol 2012 Sep 15;176(6):473-81.

© 2019 Vlachopoulos C et al. *JAMA Network Open.*

eTable 1. Cox Regression Models Predicting the Principal End Points in the SPRINT Trial and Adjusted for Model (Including Framingham Risk Score) Risk, With Estimated Pulse Wave Velocity (ePWV)

Hazard ratios	Only Model with FRS	+ ePWV
Primary outcome		
ePWV per 1-SD (1.7 m/s)		1.30 (1.17–1.43)
Chi^2	275.0	322.2
-2LogLikelihood	13099	13052
C index	0.676 (0.65–0.70)	0.683 (0.66–0.71)*
All-cause death		
ePWV per 1-SD (1.7 m/s)		1.65 (1.46–1.86)
Chi^2	142.8	205.8
-2LogLikelihood	6273	6210
C index	0.67 (0.64–0.69)	0.69 (0.66–0.72)*
Primary outcome or death		
ePWV per 1-SD (1.7 m/s)		1.36 (1.25–1.48)
Chi^2	235.0	260.1
-2LogLikelihood	9737	9712
C index	0.67 (0.65–0.69)	0.68 (0.66–0.70)*

*p<0.05 for the comparison of the "Model that includes FRS" versus the Model "+ePWV"
The numbers in parenthesis represent the 95% confidence interval.

© 2019 Vlachopoulos C et al. *JAMA Network Open.*

eTable 2. Reclassification of the Predicted Risk of Death by ePWV When Added to Model (Including FRS)

Based on the Model (including FRS)	Risk Category for All-Cause Death for the Model with FRS Additionally Considering ePWV				
	**<10%**	**10-15%**	**15-20%**	**>=20%**	**Total**
Events (n=362)					
**<10%**	280	33	5		318
**10-15%**	3	10	12	5	30
**15-20%**	1	3	1	2	7
**≥20%**	1		3	3	7
**Total**	285	46	21	10	362
Non-events (n=8,950)					
**<10%**	8,452	193	15		8,660
**10-15%**	80	83	38	12	213
**15-20%**	12	23	17	10	62
**≥20%**	1	3	7	4	15
**Total**	8,545	302	77	26	8,950

Reclassified Downward (%) 11/362 (events) and 126/8950 (non-events), Reclassified Upward (%) 57/362 (events) and 268/8950 (non-events)

The bold values represent the percentages of predicted risk in the given population. The vertical values represent the risk with our initial Model that includes Framingham risk score and the horizontal values represent the risk with our initial Model plus ePWV. The non-bold figures in the upper events section represent subjects that suffered an event during follow-up categorized based on their predicted risk by each Model, while in the lower non-events section represent subjects that were free of events during follow-up categorized based on their predicted risk by each Model.

Overall NRI=Pr(up|event) - Pr(down|event)] + [Pr(down|nonevent) - Pr(up|nonevent)] = event NRI - nonevent NRI = 46/362 - 162/8950=0.127-0.016=0.111

© 2019 Vlachopoulos C et al. *JAMA Network Open.*

eTable 3. Hazard Ratios and 95% CI for the Comparison Between Responses to Treatment Groups in the Cox Regression Survival Analysis

**All-cause death** / **Primary outcome**	**Group 1*** Standard treatment /ePWV non-responders		**Group 2** Standard treatment /ePWV responders		**Group 3** Intensive treatment /ePWV non-responders		**Group 4** Intensive treatment /ePWV responders	
**Group 1** Standard treatment/ePWV non-responders			HR=0.72 [95% CI: 0.47-1.09]	p=0.12	HR=0.54 [95% CI: 0.30-0.97]	**p=0.04**	HR=0.49 [95% CI: 0.30-0.78]	**p=0.003**
**Group 2** Standard treatment/ePWV responders	HR=0.93 [95% CI: 0.66-1.32]	p=0.69			HR=0.75 [95% CI: 0.41-1.40]	p=0.37	HR=0.68 [95% CI: 0.48-0.94]	**p=0.02**
**Group 3** Intensive treatment/ePWV non-responders	HR=0.79 [95% CI: 0.51-1.23]	p=0.30	HR=0.85 [95% CI: 0.53-1.36]	p=0.50			HR=0.90 [95% CI: 0.47-1.71]	p=0.74
**Group 4** Intensive treatment/ePWV responders	HR=0.59 [95% CI: 0.39-0.88]	**p=0.009**	HR=0.63 [95% CI: 0.48-0.83]	**p=0.001**	HR=0.74 [95% CI: 0.45-1.21]	p=0.23		

*In every box the Group where two Groups are compared the one with the smaller number is used as reference Group.

© 2019 Vlachopoulos C et al. *JAMA Network Open.*

eFigure 1. Histograms of Estimated Pulse Wave Velocity Based on Sex

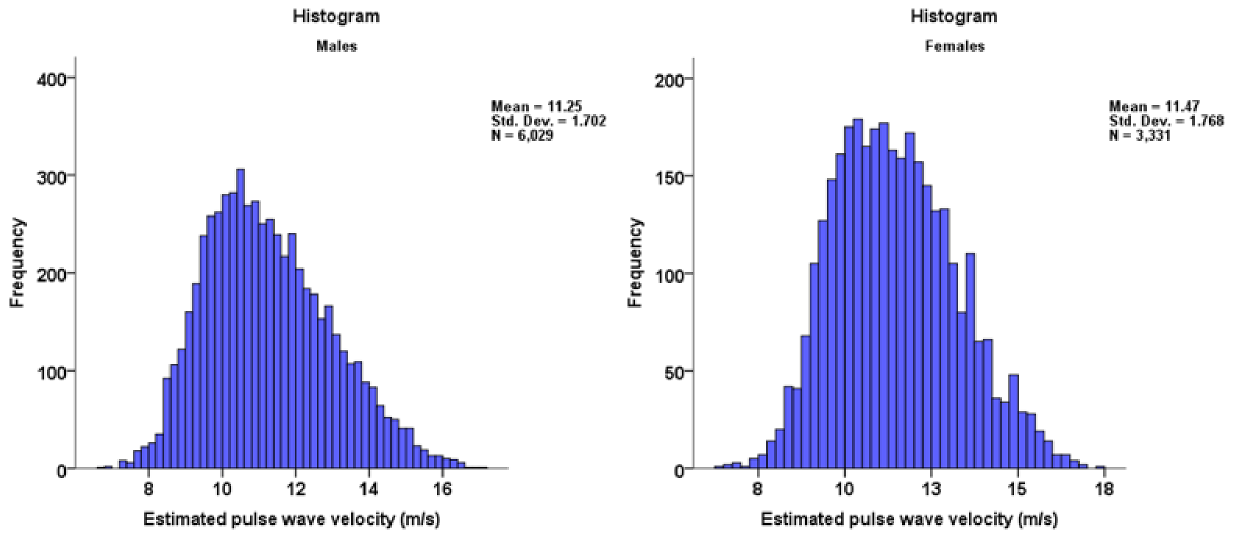

© 2019 Vlachopoulos C et al. *JAMA Network Open.*

eFigure 2. Histograms of Estimated Pulse Wave Velocity Based on Race and Age Groups

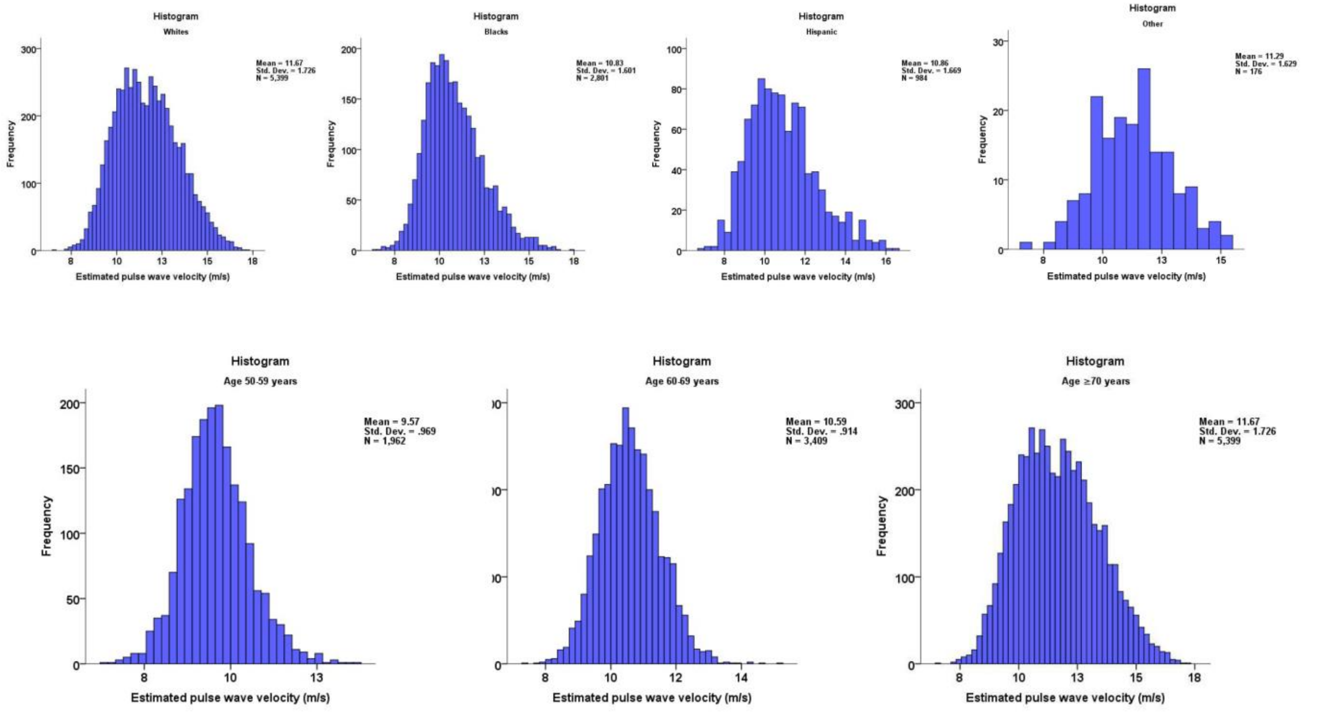

© 2019 Vlachopoulos C et al. *JAMA Network Open.*

eFigure 3. Correlation Between (A) Estimated Pulse Wave Velocity and Systolic Blood Pressure and (B) Between Their 12-Month Changes

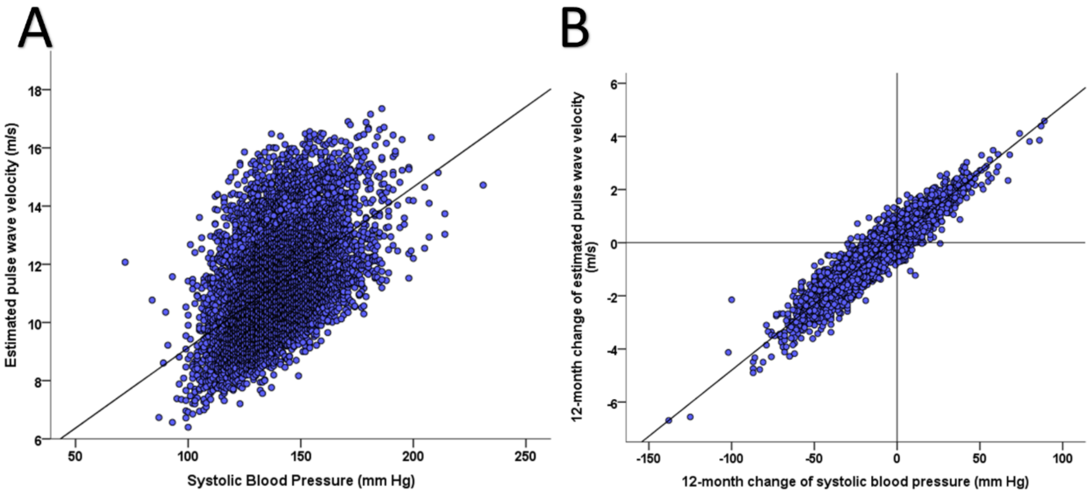

© 2019 Vlachopoulos C et al. *JAMA Network Open.*

eFigure 4. Receiver Operating Curves for the Primary Outcome and All-Cause Death

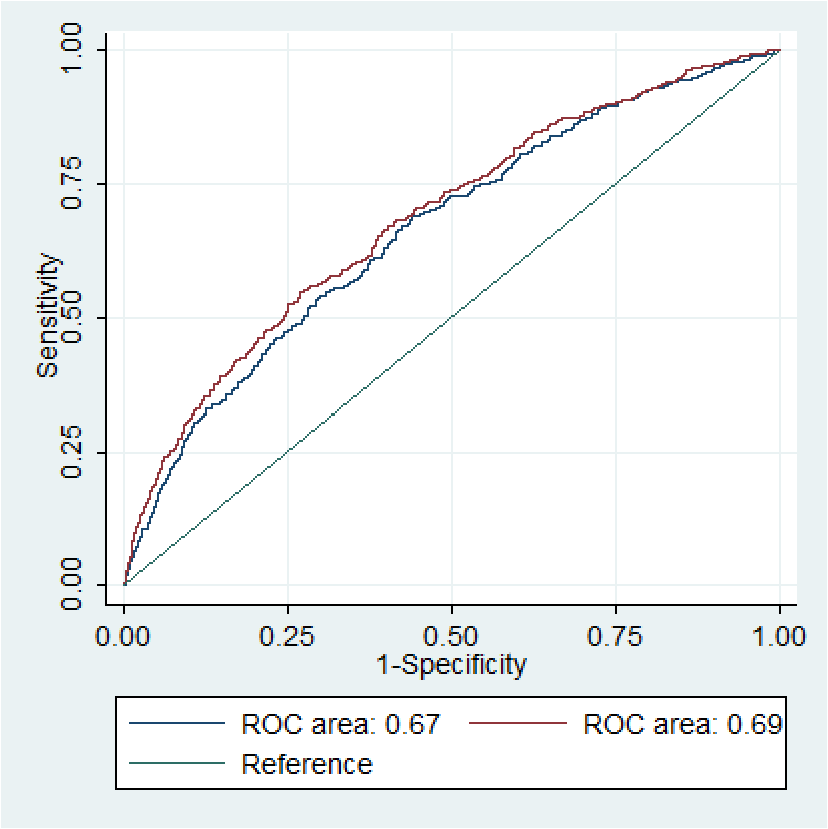

© 2019 Vlachopoulos C et al. *JAMA Network Open.*

eFigure 5. The Change of Estimated Pulse Wave Velocity at 12 Months in the Two Treatment Arms

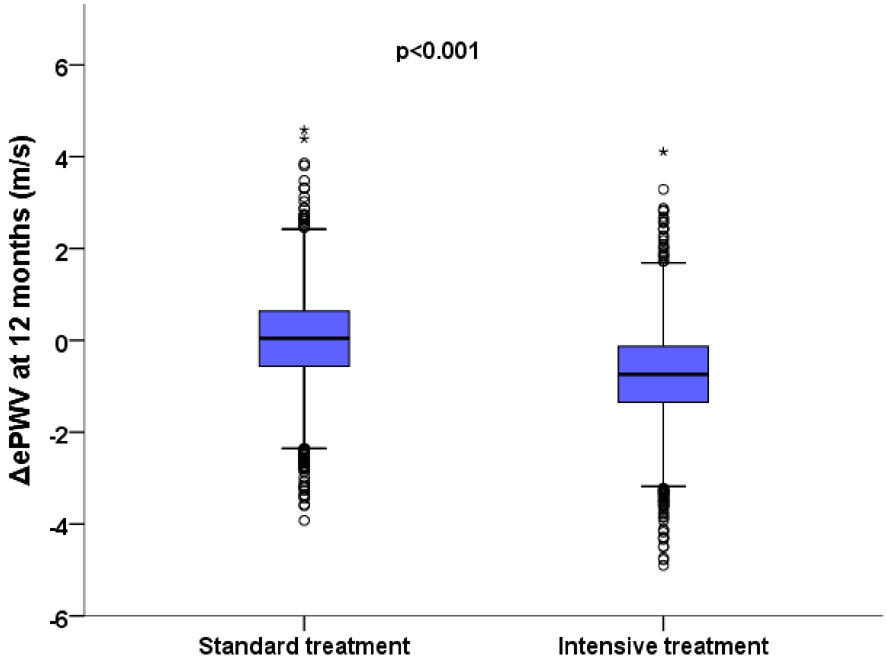

© 2019 Vlachopoulos C et al. *JAMA Network Open.*

eFigure 6. Proportion of Estimated Pulse Wave Velocity (ePWV) Responders (Green Bars) Compared to Nonresponders (Blue Bars) in the Two Treatment Arms

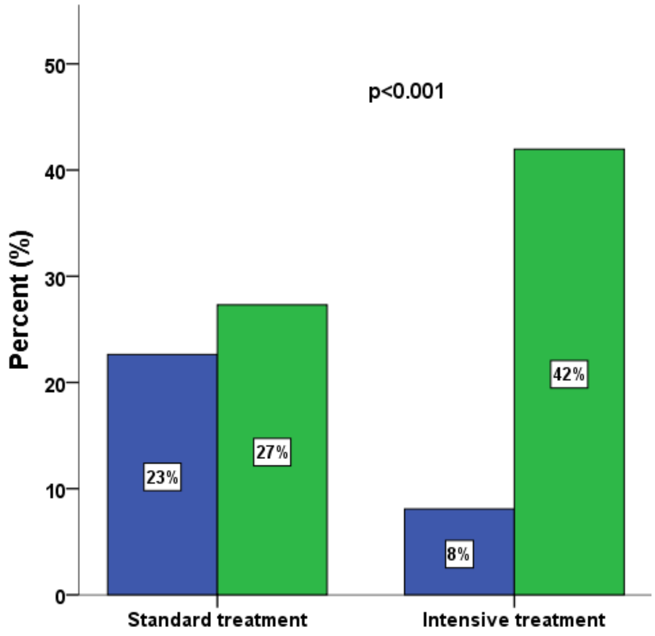

© 2019 Vlachopoulos C et al. *JAMA Network Open.*

eFigure 7. The Combined Effect of Treatment Allocation and Response of Estimated Pulse Wave Velocity (ePWV) to Treatment on the Primary Outcome
Time zero is 12 months post-randomization

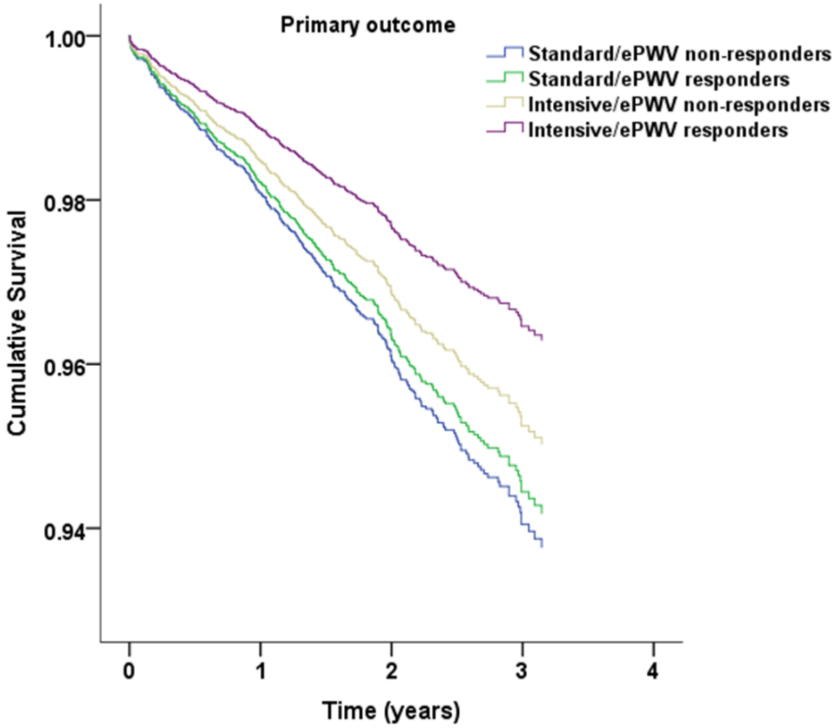

Number at risk

**Standard/ePWV non-responders**	1884	1798	1183	296
**Standard/ePWV responders**	2256	2172	1490	396
**Intensive/ePWV non-responders**	669	644	426	102
**Intensive/ePWV responders**	3486	3372	2334	649

© 2019 Vlachopoulos C et al. *JAMA Network Open.*
